# QuickStats

**Published:** 2015-03-20

**Authors:** FROM THE NATIONAL CENTER FOR HEALTH STATISTICS

**Figure:**
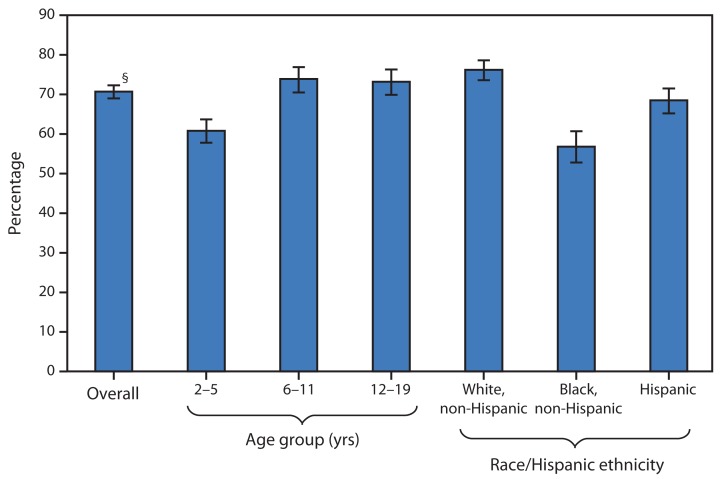
Percentage of Persons Aged 2–19 Years Who Consumed Caffeine from Food or Beverages,*^†^ by Age Group and Race/Hispanic Ethnicity — National Health and Nutrition Examination Survey, United States, 2009–2012 * The National Health and Nutrition Examination Survey collects dietary intake information using 24-hour dietary recall interviews. Day 1 24-hour recall data were used in this analysis. ^†^ Caffeine intake from foods (e.g., cookies, brownies, cakes, and candies that contain chocolate) and beverages (e.g., soda, tea, coffee, chocolate milk, and energy drinks) was calculated using the U.S. Department of Agriculture’s Food and Nutrient Database for Dietary Studies (Version 5). ^§^ 95% confidence interval.

During 2009–2012, 70.7% of persons aged 2–19 years consumed caffeine on a given day. Caffeine consumption on a given day was less common among persons aged 2–5 years (60.8%) compared with those aged 6–11 years (73.9%) and those aged 12–19 years (73.2%). The percentage of non-Hispanic black persons aged 2–19 years who consumed caffeine on a given day (56.8%) was less than that of their non-Hispanic white and Hispanic counterparts (76.2% and 68.5%, respectively). The percentage of Hispanic persons aged 2–19 years who consumed caffeine on a given day was less than that of their non-Hispanic white counterparts.

**Sources:** Ahluwalia N, Herrick K, Moshfegh A, Rybak M. Caffeine intake in children in the United States and 10-y trends: 2001–2010. Am J Clin Nutr 2014;100:1124–32.

National Health and Nutrition Examination Survey data, 2011–2012. Available at http://www.cdc.gov/nchs/nhanes.htm.

**Reported by:** Namanjeet Ahluwalia, PhD, n.ahluwalia@cdc.gov, 301-458-4372; Kirsten Herrick, PhD; Steven M. Frenk, PhD.

